# Fatty Acids as a Tool to Understand Microbial Diversity and Their Role in Food Webs of Mediterranean Temporary Ponds

**DOI:** 10.3390/molecules19055570

**Published:** 2014-04-30

**Authors:** Carla C.C.R. de Carvalho, Maria-José Caramujo

**Affiliations:** 1 IBB-Institute for Biotechnology and Bioengineering, Centre for Biological and Chemical Engineering, Department of Bioengineering, Instituto Superior Técnico, Universidade de Lisboa, Av. Rovisco Pais, Lisbon 1049-001, Portugal; 2 Centre for Environmental Biology, Faculty of Sciences, Universidade de Lisboa, Campo Grande C2, Lisbon 1749-016, Portugal; E-Mail: mariajose.caramujo@iss.it

**Keywords:** bacteria, bacterial identification, PLFA, trophic level, heterotrophic biomass, autotrophic biomass

## Abstract

Temporary Mediterranean ponds are complex ecosystems which support a high diversity of organisms that include heterotrophic microorganisms, algae, crustaceans, amphibians and higher plants, and have the potential to supply food and a resting place to migratory birds. The role of heterotrophs at the base of the food web in providing energy to the higher trophic levels was studied in temporary ponds in Central and Southern Portugal. The relative quantification of the hetero and autotrophic biomass at the base of the food web in each pond was derived from the polar fatty acid (PLFA) composition of seston through the application of the matrix factorization program CHEMTAX that used specific PLFA and their relative proportion as markers for e.g., classes of bacteria, algae and fungi. The species composition of the culturable microbial communities was identified through their fatty acid profiles. The biomass in the lower trophic level of some ponds presented an even proportion of auto to heterotrophic organisms whilst either bacteria or algae dominated in others. In a selected subset of ponds, the incorporation of bacterial fatty acids was observed to occur in potentially herbivorous zooplankton crustacean. Zooplankton consumed and incorporated bacterial fatty acids into their body tissues, including into their phospholipids, which indicates that energy of heterotrophic origin contributes to the aquatic food webs of temporary ponds.

## 1. Introduction

Temporary Mediterranean ponds are lentic ecosystems during the winter flooding period and dry during the summer when they typically support a wetland/terrestrial community. During the flood period, these ponds are likely to receive material and energy subsidies from the autochthonous source of detritus supplied by the summer vascular plant production, and may also receive allochthonous detritus either by runoff from the surrounding drainage basin or by dust deposition. The degree of autotrophy to heterotrophy may vary with the pond geochemical and environmental characteristics, yet the composition of the heterotrophic pelagic bacterial or fungal communities associated with the degradation of detritus, and the relevance of heterotrophic production for the support of zooplankon of temporary ponds (or lakes) has hardly been addressed [1,2]. Planktonic algae, including cyanobacteria and diatoms, use inorganic nutrients dissolved in water and capture CO_2_ that is converted into biomolecules. These biomolecules directly fuel the aquatic food webs or may enrich sediments with organic carbon [3] (cyanobacteria are also capable of nitrogen fixation [4]). Primary autotrophic producers have historically been considered responsible for the energy flow in many aquatic ecosystems, although the role of heterotrophy in food webs has also been recognized [5,6,7]. Organic matter originating from allochthonous sources or from excreted metabolites and/or biomass decay may be used as substrate for heterotrophic microorganisms such as bacteria and fungi [8,9], and this mass and energy may further be transferred to metazoans in the food web through the microbial loop [10,11].

Numerous studies have been devoted to the assessment of phytoplankton dietary quality and the transfer of their fatty acids (FA) to the zooplankton of lakes [12,13,14,15] while considerably fewer studies have focused on the role of FA transfer from bacteria to zooplankton, either directly or through heterotrophic protists ([16,17,18,19] and references therein). Bacteria are generally considered an unsuitable diet to crustaceans [16] although there are indications that the diversity of interactions between grazers and its bacterial food is underestimated [20]. Nevertheless, these studies concern mainly aquatic ecosystems dominated by autotrophic production and little is known of temporary aquatic systems where heterotrophic production from detritus may dominate [21]. One of the factors that may have hampered the qualitative and quantitative description of bacterial communities in aquatic studies was the nature of classical microbial tests. These tests required the isolation and subsequent culture of microorganisms, and were not adequate for the analysis of environmental samples [22]. Alternative methods are presently being applied to overcome this difficulty involving direct detection and separation of genetic and biochemical components of mixed populations to use as biomarkers. These methods include the examination of the microbial community using ribosomal RNA (16S) or corresponding DNA sequences, and phospholipid fatty acid (PLFA) analysis. Cellular membranes are mainly composed of phospholipids (PL) which are rapidly degraded to neutral lipids upon death of the organism, and thus provide valuable information of the living biomass at a given location [23]. 

In the study of microbial communities, the use of fatty acids as chemotaxonomic markers provides a faster alternative to microscopic enumerations of organisms. Chemotaxonomic markers (e.g., carotenoid and photosynthetic pigments, PLFA) have limited taxonomic resolution, yet they allow for the identification and quantification of groups of algae and bacteria. They have been successfully applied to infer phytoplankton composition and abundance in estuaries [24,25], to separate groups of algae [26] and to determine the composition and heterogeneity of the microbial community in coastal microbial mats [27] and sediments [28,29,30,31]. Data provided by pigment analysis regards mainly phototrophs while PLFA offer valuable information regarding both auto- and heterotrophs in food webs. Additionally, PLFA provide an unbiased analysis of complex microbial communities giving information on microorganisms that cannot be characterized by cultivation techniques [28]. The information provided is similar to that of phylogenetic analysis based on the sequence homology of 16S ribosomal RNA [32,33]. Individual FA can be used as biomarkers for reliable identification of microbial groups when they are present in one or few taxa, yet to extend their use for quantification purposes they must: i. be present at a nearly constant fraction of total biomass within a taxon; and ii. should be readily degraded after cell death to represent live biomass [34]. Several fatty acids have been suggested as biomarkers of certain taxa (Table 1). However, most fatty acids are hardly exclusive of an organism: e.g., cyclopropyl fatty acids are associated to anaerobic bacteria but can also be an indication of older Gram-negative bacteria because these convert monoenoic PLFA 16:1ω7c and 18:1ω7c to cy17:0 and cy19:0, respectively, as they move from logarithmic to stationary growth phase [31,35,36]. The ratios between the different fatty acids present in the microorganisms are therefore more reliable for their identification, especially if present in the phospholipids which are rapidly degraded to neutral lipids upon death of the organism. Groups of organisms can be detected by their PLFA ratios using matrix factorization programs such as CHEMTAX [37] which allows the assessment of group abundance in natural samples [24,37]

**Table 1:** Fatty acids used as taxonomic markers.

Fatty Acid	Category	Reference
*Mono-Unsaturated Fatty Acids (MUFA)*		
16:1ω7c	Bacteria	[24,26,27,38]
	Bacillariophyceae (diatoms)	
	Cyanophyceae (cyanobacteria)	
	Prymnesiophyceae	
16:1ω5c	mycorrhizal fungi	[30,39]
16:1ω8	Type I methanotrophs (gamma-proteobacteria)	[40]
18:1ω9c	Chlorophyceae (green algae)	[24,26,27,35,38]
	Cyanophyceae	
	Dinophyceae	
	Prymnesiophyceae	
	Gram-positive bacteria	
18:1ω7c	Bacillariophyceae (up to 10-fold more 18:1ω7c than 18:1ω9c)	[24,26,38]
	Cryptophyceae	
	Cyanophyceae (less amount than 18:1ω9c)	
	Prymnesiophyceae	
18:1ω7t	Gram-negative bacteria	[35]
18:1ω8	Type II methanotrophs (alpha-proteobacteria)	[39,40]
*Hydroxy substituted Fatty Acids (OH FA)*		
(e.g., 3-OH 10:0)	Gram-negative bacteria	[41]
*Cyclopropyl saturated Fatty Acids (cyFA)*		
(e.g., cy17:0, cy19:0)	Gram-negative bacteria, anaerobic bacteria	[29,31]
Iso*- and anteiso-branched Fatty Acids*		
(e.g., *i*-15:0, *a*-17:0)	Gram-positive bacteria	[29,31]
*Methyl-branched Fatty Acids (10-Me FA)*		
e.g., 10-Me 16:0	Actinomycetales (Actinobacteria)	[42]
*Polyunsaturated Fatty Acids (PUFA)*		
16:2ω7	Bacillariophyceae	[24]
16:2ω6	Chlorophyta	[24]
16:2ω4	Bacillariophyceae	[24,43]
	Prasinophyceae	
16:3ω4	Bacillariophyceae	[24,43]
16:3ω3	Chlorophyta	[24]
16:4ω3	Chlorophyceae	[24]
	Prasinophyceae	
16:4ω1	Bacillariophyceae (diatoms)	[24,43]
18:2ω6	Chlorophyta	[24,26,27,29,31,38]
	Cyanophyceae (freshwater)	
	Dinophyceae	
	Prymnesiophyceae	
	Fungi	
18:3ω6	Cyanophyceae (freshwater)	[26,31,44]
	Saprophytic fungi	
18:3ω3	Chlorophyceae	[24,26,38]
	Crypophyceae	
	Cyanophyceae	
	Dinophyceae	
	Prasinophyceae	
	Prymnesiophyceae	
18:4ω3	Various algal groups (both marine and freshwater)	[24,26,38]
18:5ω3	Dynophyceae	[24]
20:4ω6	Bacillariophyceae	[24]
	Rhodophyceae	
20:5ω3	Bacillariophyceae	[24,38]
	Cryptophyceae	
	Dinophyceae	
	Pavlovophyceae	
	Rhodophyceae	
22:5ω3	Bacillariophyceae	[24]
	Cryptophyceae	
	Prasinophyceae	
22:6ω3	Bacillariophyceae	[24,26,38]
	Cryptophyceae	
	Dinophyceae	
	Haptophyta (Prymnesiophyceae and Pavlovophyceae)	

Data on the FA composition of organisms at the base of the aquatic food web may further be used to disclose trophic connections with metazoans. FA molecules have the particularity of presenting specificity for some groups of organisms which, allied with the conservative transfer of the dietary FA composition to the consumers, enables their use as trophic biomarkers [38]. Thus FA may be used as versatile tools in trophic transfer studies to disclose dietary relationships, and to understand aquatic food webs structure and functioning [45]. Direct incorporation of dietary FA into the lipid reserves of organisms, especially into triacylglycerides (TAG), has been demonstrated (see [38]) although FA may be potentially modified by animals during metabolism and transportation, FA may be potentially modified by animals which may have a significant impact on their utility as trophic tracers. Fatty acids have been a focus of increasing interest in the study of aquatic food webs [46,47]. This interest largely derives from the recognition of the importance of certain types of FA such as ω3 polyunsaturated FA (PUFA) for the growth of herbivorous zooplankton [14,48] and ultimately for the health of juvenile fish and the success of fisheries [49,50].

The objectives of the present study were to (i) explore the use of PLFA to determine the degree of heterotrophy *vs.* autotrophy in temporary ponds, and (ii) to inspect the incorporation of FA of heterotrophic origin into the tissues of crustacean zooplankon. Although PLFA have become well-established chemotaxonomic markers of bacteria [23,24 and references therein], the availability of bacterial PLFA composition in literature is still scarce. Therefore, we first assessed the PLFA composition of bacteria present in temporary ponds. Through cultivation-based techniques, bacteria were isolated and total fatty acid profile of each isolate was used for its identification. After the identification of bacteria, the PLFA composition of the cultivated bacterial and fungal groups, and that of algae obtained from literature, were used to derive group and abundance of seston in terms of classes of autotrophs and heterotrophs. After determining the availability of group composition in the seston, we inspected how the FA originating from heterotrophs and autotrophs were incorporated into the tissues of crustacean zooplankon.

## 2. Results and Discussion

**Figure 1:**
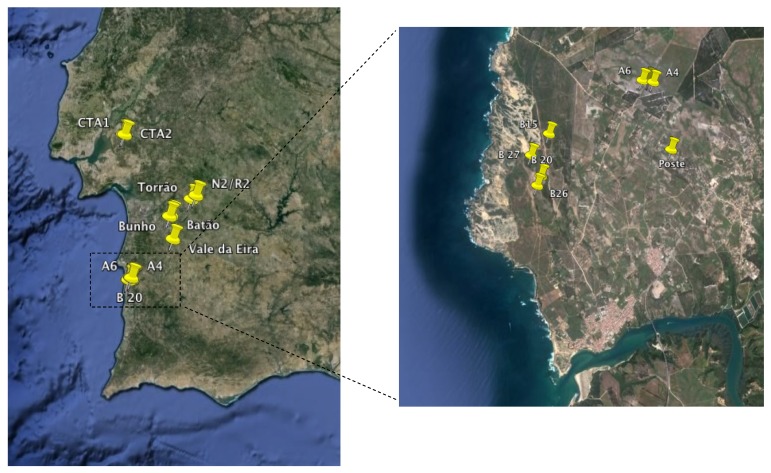
Location of the temporary ponds studied in southern Portugal (Google Earth v7.1. DigitalGlobe 2013).

The temporary ponds studied were located near the southwest coast of Portugal, between N 38° 46.745' and N 37° 45.411' (Figure 1). The identity code, geographic coordinates and physical properties of the water of each pond are listed in Table 2. All ponds were sampled twice at one week interval between late January and early February 2010, except CTA ponds which were sampled only once in May 2010. Human impacts on the ponds and surrounding fields were restricted to sheep herding and physical trampling of pond margins during the dry periods with the exception of Batao 1 and Poste which are surrounded by agricultural fields.

**Table 2:** Code, location, physical data and biomass of chlorophyll-*a* present in the sampling sites. FFR of Alcochete – Field Firing Range of Alcochete; PNSACV – Natural Park of the Southwest Alentejo and Vicentina Coast in Portugal.

Code	Location	GPS coordinates	Depth	Temperature	Conductivity	Salinity	Chlorophyll-*a*
			m	°C	μS	ppt	μg L^−1^
CTA1	FFR of Alcochete	N 38° 46.745' W 8° 51.621'	0.4	17.4	177.7	0.1	40.8
CTA2	FFR of Alcochete	N 38° 46.687' W 8° 50.979'	0.4	17.8	112	0.1	47.7
CTA3	FFR of Alcochete	N 38° 46.634' W 8° 50.238'	0.4	15.8	265.1	0.1	38.2
CTA5	FFR of Alcochete	N 38° 46.363' W 8° 47.374'	0.6	16.6	153	0.1	4.4
CTA6	FFR of Alcochete	N 38° 46.363' W 8° 47.374'	1.1	16.6	135.3	0.1	14.6
N2/R2	Torrão	N 38° 21.234' W 8° 11.959'	0.5	13.7	116.6	0.1	3.9
TORRAO	Torrão	N 38° 19.670' W 8° 14.587'	1.2	13	54.4	0	4.1
BATAO 1	Batão	N 38° 12.863' W 8° 23.675’	0.6	12.4	360	0.1	78.3
BATAO 2	Batão	N 38° 12.562' W 8° 24.791'	0.6	12.3	297	0.1	28.6
BATAO 3	Batão	N 38° 12.870' W 8° 25.270'	0.6	12.4	281	0.1	7.1
BUNHO	Bunho	N 38° 12.287' W 8° 26.168'	0.6	17.4	111	0.1	27.1
V EIRA	V. da Eira	N 38° 2.718' W 8° 24.193'	0.6	14.6	596	0.1	23.9
A5	PNSACV, Sector A	N 37° 46.147' W 8° 46.335'	0.5	11.6	158.1	0.1	1.3
A4	PNSACV, Sector A	N 37° 46.128' W 8° 46.277'	0.7	13.9	119.2	0.1	0.7
A6	PNSACV, Sector A	N 37° 46.147' W 8° 46.410'	0.7	12.1	167.2	0.1	2.8
B15	PNSACV, Sector B	N 37° 45.570' W 8° 47.666'	0.9	14.2	175	0.1	1.4
B20	PNSACV, Sector B	N 37° 45.124' W 8° 47.769'	0.7	13.8	542	0.1	2.2
B26	PNSACV, Sector B	N 37° 45.018' W 8° 47.820'	0.2	13.9	715	0.1	3.3
B27	PNSACV, Sector B	N 37° 44.997' W 8° 47.903'	0.8	13.3	621	0.1	0.5
POSTE	PNSACV, Sector A	N 37° 45.411' W 8° 46.017'	0.2	14	139.2	0.1	0.9

**Figure 2:**
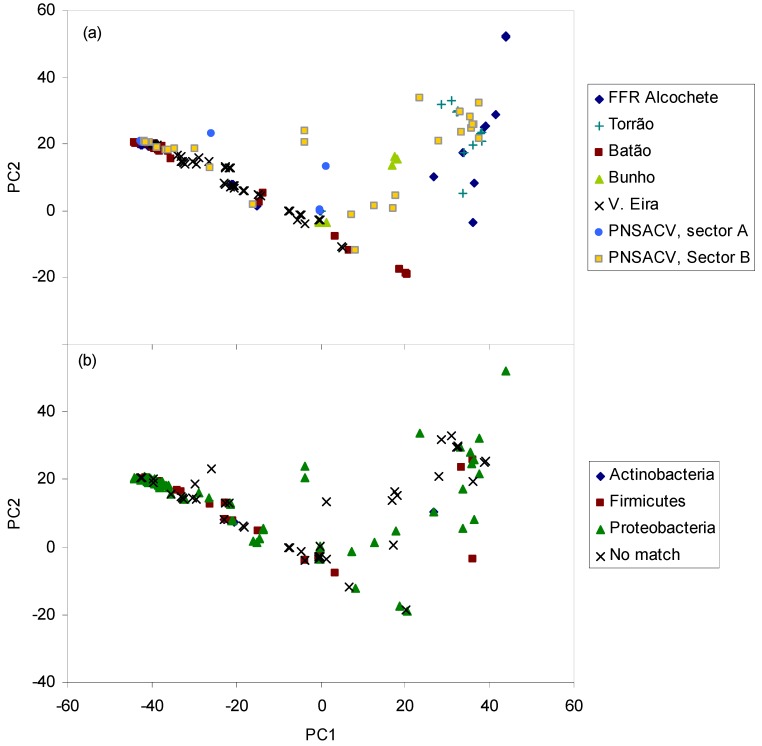
Scores of the sampled ponds (upper panel) and phyla of bacterial identified in the ponds (lower panel) along the first two axes resulting from PCA of fatty acid composition of bacterial isolates.

In lakes and ponds, bacteria have a range of habitats including the air-water interface, the benthic and anoxic zones [51]. Although growth-cultivation techniques may not represent the entire microbial community, because some microorganisms do not grown under laboratorial conditions [52], successful growth of a given strain is an unequivocal evidence of its presence. A cultivation-based characterization of the microbial community, in particular of the bacterial community, can be accomplished by the analysis of the FA profile [53,54]. In fact, the fatty acid profile of microorganisms can function as a fingerprint for their identification. It is an accurate, high-throughput method with a low cost per sample [55]. For this, the cells are grown in defined medium (e.g., trypticase soy agar, blood agar or Middlebrook agar) at a given temperature and collected after a certain defined period because the fatty acid composition changes in response to both growth temperature and age of the culture. Even when the FA profile is from an unknown microorganism, statistical techniques (e.g., hierarchical clustering or principal components analysis) may be applied to disclose the proximity of the unidentified species to a given microbial taxa (Figure 2).

In the present study, inoculations (100 µL of sample) were initially done on trypticase soy agar (TSA) plates and, simultaneously, on 10- and 100-fold diluted TSA plates to facilitate the isolation of bacteria from nutrient depleted environments. All colonies grown in both diluted and standard TSA were then plated on new TSA plates where the cells were grown at 30 °C during 24h. After 24h±1h, the cells were collected and each bacterium was identified applying the method of the Sherlock^®^ Microbial Identification System (MIDI, Inc., Newark, DE, USA) using the bacterium’s unique fatty acid profile. The bacteria present in the samples collected in the temporary water ponds belonged to the phyla Actinobacteria, Bacteroidetes, Firmicutes and Proteobacteria. The species data presented refers to the sum of species present in each pond sampled twice at one week interval. All other data values are the average of the two sampling dates. The species belonging to Actinobacteria were *Rhodococcus equi*, *Micrococcus luteus* and *Kocuria rhizophila*. A single species of *Sphingobacterium spiritivorum* of the phylum Bacteroidetes was isolated and so this phylum was disregarded when evaluating the importance of bacteria in the ponds. The phylum Firmicutes was mainly represented by *Bacillus* species although other species were also isolated from samples collected both in the water column and sediments: *B. cereus*, *B. marisflavi*, *B. megaterium*, *B. pumilus* and *B. thuringiensis*, *Brevibacillus laterosporus* and *Staphylococcus xylosus*. However, most of the bacteria isolated belonged to the phylum Proteobacteria which contain enterobacteriaceae (Figure 2b). Among the species isolated were *Achromobacter xylosoxidans*, *Aeromonas caviae*, *A. hydrophila*, *Alcaligenes faecalis*, *Comamonas terrigena*, *Edwardsiella tarda*, *Enterobacter aerogenes, Escherichia coli*, *Ewingella americana*, *Kluyvera intermedia*, *Neisseria sicca*, *Pantoea agglomerans*, *Pseudomonas fluorescens*, *P. putida*, *P. syringae*, *Serratia liquefaciens*, *S. odorifera*, *Stenotrophomonas maltophilia*, *Yersinia intermedia*, *Y. kristensenii* and *Yokenella regensburgei*. 

**Figure 3:**
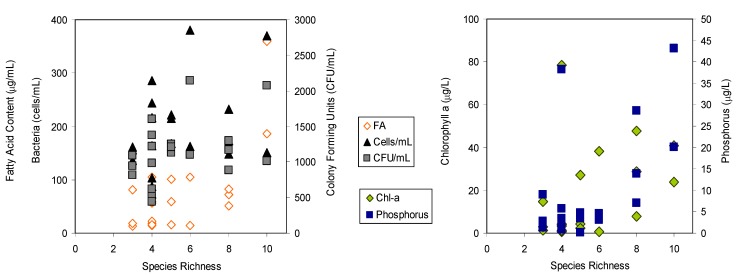
Relationships between bacterial species diversity as the total number of species identified in each pond (species richness) using cultivation techniques and (left panel) colony forming units (CFU/mL), bacterial cell abundance determined by microscopy (cells/mL) and total FA content of seston; and (right panel) chlorophyll-*a* and dissolved phosphorus (P) concentration in pond water.

Heterotrophic organisms may dominate microbial communities in vernal pools [56] and Mediterranean streams which suffer extreme seasonality (droughts and floods) [57]. These groups include species efficient in oxidizing ammonia, in nutrient assimilation and substrate mineralization, specially when organic and humic compounds are present in abundance and/or in systems with hydrological fluctuations [57,58,59]. Furthermore, temporary water ponds offer refuges for birds and serve as water supply for domestic animals such as sheep and cows. Storks, flocks of sheep and cows were observed at the ponds at PNSACV (both sectors A and B), N2R2 and Torrao which presented mainly proteobacteria (Figure 2). The animals should thus be a considerable source of bacteria which is corroborated by the high number of enteric species isolated. Nevertheless, the number of colony forming units per mL (CFU/mL) ranged between 0.44–2.44 × 10^3^ CFU/mL in the water column (Figure 3) and 0.65–2.81 × 10^3^ CFU/mL in the sediments (data not shown). The number of CFUs were 10%–25% lower than direct microscopic counts of bacteria in samples (Figure 3). It was expected that direct microscopic counts would provide significantly higher number of cells than CFUs since direct counts include viable, viable but non-culturable, and even non-viable bacterial cells. The present results thus indicate that most of the bacterial population was in fact in a viable state. It has been suggested that most cells of the bacterioplankton do not produce screening pigments as response to solar radiation (which is high in shallow temporary ponds in Southern Portugal) but overcome the stress by fast cell division and effective repair mechanisms [60], which could explain the high percentage of viable cells. The obtained bacterial numbers are lower than those obtained by previous studies that reported 10^4^ CFU/mL in the pond water and 10^7^–10^8^ per gram of soil in drying or dried ponds [6], and 9.6 × 10^4^–1.3 × 10^7^ cells/mL in oligotrophic lakes [61]. The total organic carbon available in the water of all ponds in the present study was below 2.27 ppm whilst the available nitrate and ammonium were below 1.55 and 1.79 mg/L, respectively (data not shown), and dissolved phosphorus (dP, Figure 3) was below 20 μg/L in most ponds (except Batao 1, Batao 2 and CTA1). The nutrient depleted environment of most temporary ponds could have contributed to the low microbial numbers of bacteria observed. It has been postulated that the bacterioplankton is mainly determined by substrate availability and grazing [62] as well as environmental conditions [63]. The samples in the present study were collected in Winter (with the exception of CTA ponds), and both nutrient depletion conditions and low temperature should have hampered bacterial growth in the ponds. 

Fungi were also observed on the TSA plates inoculated with 100 μL of sample from each pond, although at low numbers (representing at the most 1%–5% of the microbial community; data not shown). Both algae and fungi on the samples were identified by direct observation and microscopy images using published identification keys and taxonomy literature [64,65,66]. The observed fungal species belong to the phyla ascomycota, zygomycota and basidiomycota. Almost one-third of freshwater ascomycetes have been isolated from lakes and/or ponds [67] and some zygomycetes which are predators of amoebae, rotifers and nematodes are also common in freshwater pools [68]. Ascomycetes and basidiomycetes are usually associated with decaying leaves in freshwater environments [67,69].

The total FA composition of isolates that was required for the identification of the microbial community in the temporary ponds provided information regarding which biomarkers and FA ratios could be used to identify bacterial groups. However, this information had to be refined, and the PLFA composition of isolates was analysed, to enable comparisons with FA originating from living organisms (*i.e*., PLFA) in the seston of field samples. It has been shown that changes in the microbial community structure can be detected by modifications in the PLFA patterns in sediments and soil [33,70,71], vernal pools [56] and aquatic environments [27,38]. 

The PLFA of each species of bacteria and fungi were thus used to determine generic ratios of fatty acids representing each phylum present in the temporary ponds (Table 3). The PLFA composition of the algae present was the combination to that of published studies [24,26,38]. Bacterial phyla presented *iso*- and *anteiso*-methyl branched fatty acids not present in either fungi or algae and distinct ratios of other common fatty acids (Table 3). Fungi presented higher amounts of 18:1ω9c than any other microbial group. Green algae and cyanobacteria differed from the others by producing higher amounts of 18:3ω3, whilst diatoms produced larger quantities of 20:5ω3 and 22:6ω3 and also produced 16:2ω4 and 16:3ω4. The PLFA ratio matrix present in Table 3 was used as input in the matrix factorization program CHEMTAX [37] to determine the group abundance from the PLFA composition of each temporary pond (determined by PLFA extraction from filters containing the biomass from filtered water). This program, initially created to determine group abundances using pigment information [37,72,73,74], has been successfully used to determine group abundances using also PLFA data [24,27].

The program CHEMTAX adapts the input ratio and determines iteratively which ratios best fit the data. The output PLFA ratio matrix is also presented in Table 3. CHEMTAX only adapted the ratio of both *iso* and *anteiso*-15:0 in proteobacteria and of 16:1ω7c and 18:1ω9c in basidiomycetes to reflect the variations observed in the temporary ponds. The data was analyzed by CHEMTAX per pond location so that only phyla present in each location was used. However, when all data were analyzed simultaneously, the outcome was basically the same as when data was split and analysed by location (data not shown). Since the CHEMTAX is known to be sensitive to the values of the initial ratio matrix, ratio optimization was tested according to previous studies [73,75] but similar results such as those presented in Table 3 were also attained (data not shown). Since these results were obtained after adjustment of each ratio up to ±50%, as suggested, this gives an indication of the stability of the results.

**Figure 4:**
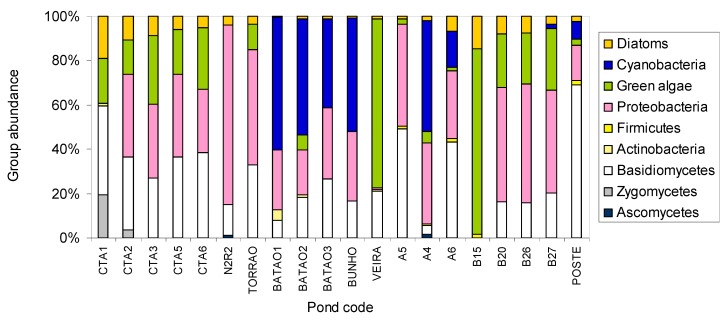
Relative abundance of autotrophic (diatoms, cyanobacteria and green algae) and heterotrophic (proteobacteria, firmicutes, actinobacteria, basidiomycetes, zygomycetes and ascomycetes) biomass in temporary ponds in southwest Portugal.

Group abundance according to CHEMTAX results indicate that ponds N2R2, Torrao, A5 and Poste contained mainly (above 87%) heterotrophic biomass whilst in ponds V EIRA and B15 the energy at the basis of the aquatic food web was largely derived from autotrophs (Figure 4). In the former ponds, the majority of the microorganisms belonged to proteobacteria and basidiomycetes whilst on the latter ponds, green algae accounted for ca*.* 80% of the biomass. Around 50% of the biomass in the ponds in Batão and Bunho belonged to cyanobacteria, which were also present in the same percentage in pond A4 at PNSACV, sector A. The PLFA analysis indicate that the temporary ponds located at the FFR of Alcochete (CTA2-5) and at PNSACV, Sector B (B20, B26 and B27) contained over 30% of proteobacteria, *ca.* 20% green algae and 4%–10% diatoms. However, ponds at FFR of Alcochete presented larger amounts of fungi (average 34.6%) than those at PNSACV (average 17.4%). At CTA1 59.4% of the biomass were fungi, with basidiomycetes accounting for 39.8%. It has been postulated that increased fungi abundance when compared to bacteria is related to reduced rates of nutrient cycling and increased retention of C and N [76,77]. Nevertheless, the fact that CTA ponds were sampled in spring may have contributed to the larger growth of fungi relative to bacteria in these ponds.

According to the CHEMTAX group abundance, proteobacteria was the dominant bacterial phylum in all ponds, with the exception of CTA1 and B15 where actinobacteria and firmicutes were the predominant phyla (Figure 4). This is in accordance with the results obtained by growth cultivation and bacteria identification (Figure 2). β-Subclass of proteobacteria were also frequently identified in sediments and overlying water body of freshwater lakes by SSU rRNA gene analysis [78] and fluorescence *in situ* hybridization [79].

**Table 3:** Average PLFA composition (referred to 16:0) for bacteria, algae and fungi isolated in the samples collected in the studied temporary ponds and cultured under standard conditions. PLFA present at a very low concentration (less than 1%) or occurring in single species were omitted.

Class/	Ascomycetes	Zygomycetes	Basidiomycetes	Actinobacteria	Firmicutes	Proteobacteria	Green algae	Cyanobacteria	Diatoms
Fatty acid									
Input matrix ratio									
12:00	0.06	0.03	0.04	0.01	0.05	0.03			
14:00	0.09	0.06	0.05	0.28	0.31	0.07	0.06	0.03	0.99
15:00				0.11	0.1	0.02			
15:0 anteiso			1.13	3.88	0.16			
15:0 iso				0.78	2.25	0.1			
16:00	1	1	1	1	1	1	1	1	1
16:1ω7c	1.66	0.16	0.1	0.01	0.05	0.02	0.21	0.2	1.93
16:1ω9c								0.29	0.02
16:2ω4									0.39
16:3ω4									0.69
17:00	0.04	0.03	0.03	0.04	0.02	0.02			
17:0 iso				0.13	0.3	0.03			
18:00	0.24	0.2	0.17	0.07	0.06	0.05	0.09	0.05	0.07
18:1ω9c	2.87	1.98	1.1	0.64	0.04	0.01	0.3	0.38	0.07
18:1ω7c	0.13	0.09	0.11				0.09	0.15	0.16
18:2ω6c	0.94	0.66	3.28				0.38	0.45	0.08
18:3ω3	0.07	0.49	0.6				2.27	0.83	0.03
20:5ω3							0.01		1.76
22:6ω3									0.19
Output matrix ratio									
12:00	0.06	0.03	0.04	0.01	0.05	0.03			
14:00	0.09	0.06	0.05	0.28	0.31	0.07	0.06	0.03	0.99
15:00				0.11	0.1	0.02			
15:0 anteiso			1.13	3.88	0.09			
15:0 iso				0.78	2.25	0.07			
16:0	1	1	1	1	1	1	1	1	1
16:1ω7c	1.66	0.16	0.05	0.01	0.05	0.02	0.21	0.2	1.93
16:1ω9c								0.29	0.02
16:2ω4									0.39
16:3ω4									0.69
17:00	0.04	0.03	0.03	0.04	0.02	0.02			
17:0 iso				0.13	0.3	0.03			
18:00	0.24	0.2	0.17	0.07	0.06	0.05	0.09	0.05	0.07
18:1ω9c	2.87	1.98	0.88	0.64	0.04	0.01	0.3	0.38	0.07
18:1ω7c	0.13	0.09	0.11				0.09	0.15	0.16
18:2ω6c	0.94	0.66	3.28				0.38	0.45	0.08
18:3ω3	0.07	0.49	0.6				2.27	0.83	0.03
20:5ω3							0.01		1.76
22:6ω3									0.19

Light reaches the bottom in all the sampled shallow ponds, and it is not considered to be a limiting factor of photosynthesis. As noted by Roberts and Howarth [80], the rates of both bacterial and phytoplankton respiration increase when nutrient availability increases although the highest relative contribution of bacterial respiration occurs under the most oligotrophic conditions (total phosphorus < 24 µg L^−1^, [81]). Under oligotrophic conditions bacteria are better competitors for nutrients that limit both autotrophs and heterotrophs, but as nutrient availability increases, conditions become increasingly favourable for larger celled phytoplankton that have higher maximal nutrient uptake rates. In the present study, chlorophyll-*a* biomass is significantly related to total phosphorus (dP) indicating that phytoplankton is favoured by the increase in nutrient availability (Spearman’s r = 0.605, *p* = 0.01). The increase in the total FA biomass in the seston < 125 μm of the ponds is significantly correlated to the increase of chlorophyll-*a* biomass (Spearman’s r = 0.749, *p* = 0.01), yet it is not significantly related to the increase in the proportion of autotrophs in ponds (r = 0.164, *p* > 0.05) which suggests that an increase of heterotrophic biomass must have also occurred.

The application of CHEMTAX (like other type of factorial analysis) to derive the potential group abundance of organisms at the base of the food webs assumed that only the types of organisms observed by microscopy were present in the plankton, *i.e*., algae, bacteria and fungi. The presence of heterotrophic protists in the plankton was rarely observed in samples, and we considered that their abundance was sufficiently low at the time of sampling to prevent interference from their FA composition in the calculation of abundances of other organisms. Data on the FA composition of heterotrophic protists are scarce, especially for freshwater protists [82,83,84] yet, it has been suggested that in the natural environment the main factors influencing the FA content of some freshwater protists (e.g., flagelates) are the FA contents of picoplankton prey and of dissolved organic matter [83,85]. Thus the composition of protist FA would have considerably reflected their food source and thus the class of organisms at the basal level of the food web. 

**Figure 5:**
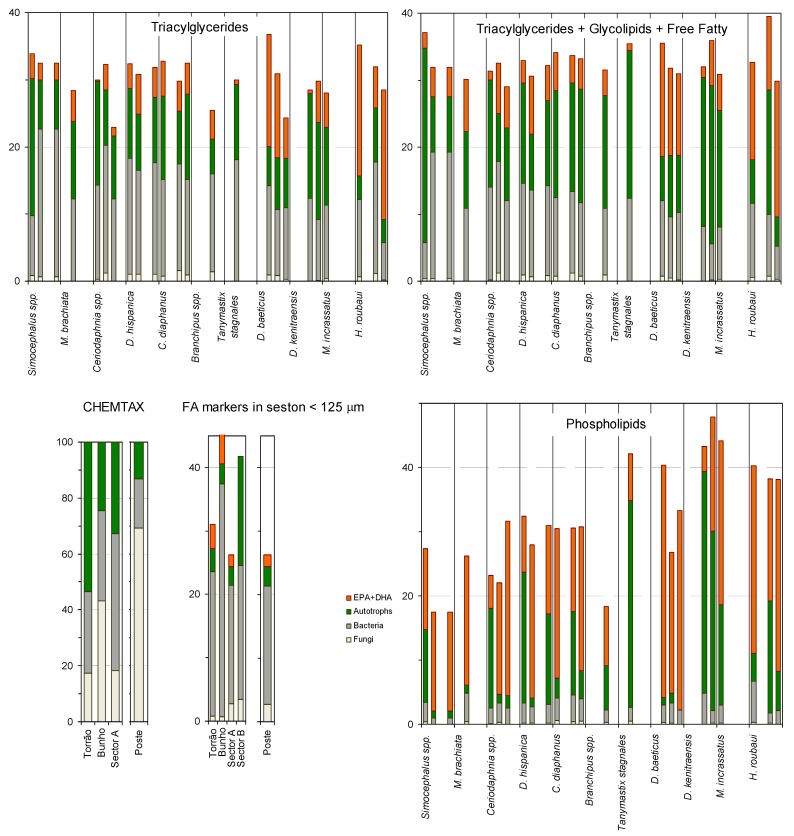
Relative abundance of the groups of autotrophs, bacteria and fungi in pond seston calculated by CHEMTAX, and calculated from the sum of FA markers of each group (lower left hand side panels). The content of the same FA markers in zooplankton was calculated in the neutral lipids of zooplankton (upper left panel), in the sum of their neutral lipids plus acetone mobile (glycolipids) and free fatty acids (upper right panel) and in zooplankton phospholipids (lower right panel). FA marker of fungi is 16:1ω5, FA markers for bacteria are 15:0, *ante-* and *iso*- FA, hydroxy substituted FA, cyclopropyl saturated FA and 18:1ω8, and FA markers of autotrophs are C16 PUFA and C18 PUFA. *Tanymastix stagnales* was present only in pond “Poste”. The other zooplankton were collected in a set of 4 ponds and are depicted in the order (from left to right) Torrao, Bunho, A6 and in ponds B20-B27. Please note the different YY axis scales.

In order to explore the possible utilization of heterotrophic biomass by grazers, the relative proportion of fatty acids of fungal and bacterial origin in the seston were compared to those in the potentially herbivore zooplankton (Figure 5). The set of ponds chosen for comparison of seston FA *versus* potentially herbivorous species FA were Torrao, Bunho, A6, B27 and Poste. According to the results of CHEMTAX analysis (Figure 4), autotrophs and heterotrophs were represented in similar proportions in the pond Bunho while in all other ponds the heterotrophs were in higher proportion than autotrophs. When only FA specific of bacteria or algae are considered, Bunho has the higher proportion of bacterial FA and since we inspected these markers in the zooplankton lipid reserves, it was this latter proportion that was taken into consideration (Figure 5). Nevertheless we could not establish a direct relationship between FA in the reserve lipids (triacylglycerides) of consumers in each pond and their abundance in the seston of the corresponding pond. Bacterial fatty acids are indeed deposited into the consumers triacylglycerides, and to a lesser extent, into other lipid classes including the phospholipids which reflect the structural requirements of membranes and regulate membrane fluidity (Figure 5 [86,87,88]).

Small herbivorous branchiopods (cladoceran *Simocephalus* sp., *M. brachiata*, *Ceriodaphnia* sp. and *D. hispanica*) have FA profiles of all lipid classes more similar to those of large herbivorous branchiopods (*C. diaphanus* and *B. cortesi*) than to those of copepods of similar size as previously observed for cladocerans of various sizes and copepods [89]. *Daphnia*, other cladocerans and Anostraca branchiopods such as *Branchipus* have the ability to directly feed on small sized plankton, including bacteria [90] while other zooplankton may require repackaging of picoplankton or bacteria through protists because of their filter apparatus. Nevertheless, bacterial fatty acids were observed in the reserve lipids of all zooplankton analysed in this study. In the zooplankton of temporary ponds, it is also apparent that not all “essential fatty acids” are retained similarly as previously noted for lakes and the marine environment [91]. FA of autotroph origin and EPA and DHA are retained in the triacylglycerids of zooplakton at a higher proportion than those available in the seston (Tables S1–S5). It is likely that zooplankton retains and accumulates preferentially the FA most important for their metabolism instead of retaining them in the proportions available in the potential food. Lee *et al.* (1971) in their pivotal studies observed that the composition of copepods closely matched the FA composition of their diets noting that the correspondence between diet and copepod FA increased when food concentrations were higher [92]. Similarly, Kainz *et al.* examined the accumulation of essential fatty acids (EFA) in different zooplankton size classes for a series of lakes and found that all zooplankton size classes accumulated 2–4 fold more essential fatty acids than their proportion in the seston [93]. Crustaceans of temporary environments are likely to have evolved with the ability to retain the more nutritiously important FA from the generally poor quality food available in these rather unpredictable habitats [2]. Thus the proportion of prey FA may be largely attenuated by the consumer metabolism when FA are deposited into the consumer tissues [45]. It is known that the FA composition of the storage lipid fraction in marine copepods is influenced by their diet, and it is generally believed that dietary FA are incorporated, rather unmodified, into these lipids [38,92]. Nevertheless, copepods from ponds, *Daphnia,* and *Simocephalus* were shown to elongate/desaturate 18:3ω3 to 20:5ω3 [45,84,85,94] although it is not clear if they have the ability to deposit those modified FA into their reserve lipids (triacylglycerides) or only into other lipid classes.

Other factors influencing the lack of match between the FA composition of reserve lipids of zooplankton and seston may be related to their utilization of other food sources in association to seston. *Simocephalus*, although a component of plankton in temporary ponds, are mainly benthic and feed also on detritus [84]. Consequently, the observed high content of bacterial FA in the reserve lipids of *Simocephalus* from Bunho and Sector B pond (Figure 5) may derive from benthic bacteria or bacteria attached to submerged macrophytes. The calanoid copepod *H. roubaui* was also observed to be able to attack and feed on other zooplankton which may be responsible for the high proportion of EPA and DHA in its reserve lipids (Figure 5).

**Figure 6:**
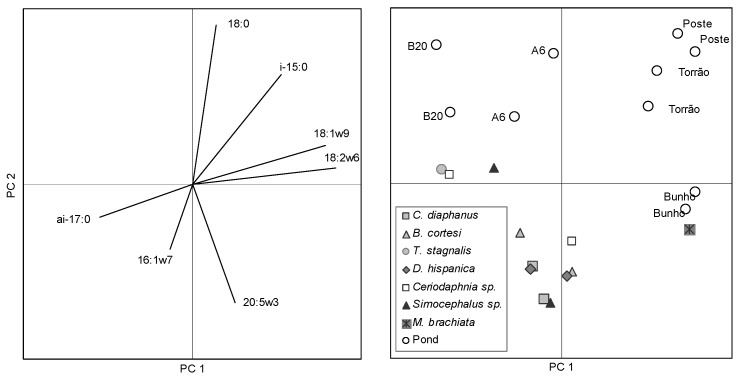
Factor loadings (left handside panel) of the fatty acid (FA) variables on the first two principal components (PCs) resulting from the application of PCA to FA weight percentage in the seston (<125 μm particle size) and in the apolar lipid fraction (reserve lipids) of pond zooplankton. Eigenvalues for PC1 = 2.6; PC2 = 1.7. Scores (right handside panel) of the ponds and zooplankton in the plane created by the first two axes resulting from PCA of fatty acid variables.

**Figure 7:**
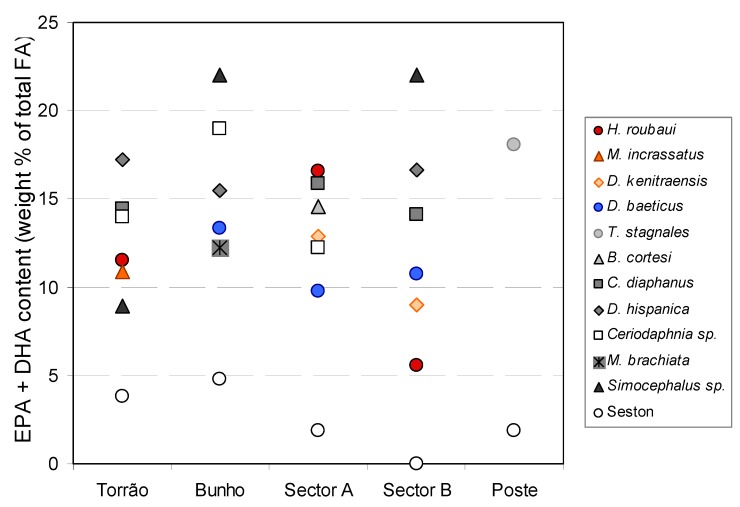
Variation of EPA and DHA content in zooplankton reserve lipids relative to seston content (<125 μm particle size) in the corresponding pond of origin (open circles).

The lack of a match between potential diet and FA zooplankton composition can be observed through the application of multivariate analysis to FA data (Figure 6). The first two principal factors or components (PC1 and PC2) obtained from the application of PCA to the fatty acid composition of pond seston (<125 μm particle size) and in the reserve lipids (triacylglycerides) of zooplankton explained 61.4% of data variability. The relative contribution of each original variable to the PCs (loadings) is directly proportional to the projection of the length of the variable vector on the PCs (Figure 6). PC1, explaining 37.6% of the total variance, is mainly a function of 18:2ω6 and *anteiso*-17:0 contributing in opposite directions. PC2 explained 23.8% of total data variance and had as major contributors 18:0 and 20:5ω3 (Figure 6). The position of Bunho plotted on the plane created by the first two PCs reflects its high seston content of 18:2ω6 which was translated in a high biomass of cyanobacteria calculated by CHEMTAX. The ponds were mainly plotted according to their content in 18:0 and either their seston had more 18:2ω6 and 18:1ω9 (Torrao and Poste) or *anteiso*-17:0 (sector A and B ponds). Only the cladoceran *Moina brachiata* exhibits proximity between the FA composition of its triacylglycerides and that of pond seston. Selective retention or bioconversion of FA acids seems to have contributed for the lack of match between the FA content of crustacean lipid reserves and that of seston as indicated by the clear higher EPA and DHA content of crustacea relative to that of seston (Figure 7). 

The diversity of interactions between grazers and its bacterial food may still be largely underestimated. Quality limitations of bacteria may extend to sterols as indicated by *Daphnia* species that have the ability to feed on heterotrophic bacteria, and yet are limited by sterols and not PUFA in their use of bacteria as a carbon source [20].

## 3. Experimental

### 3.1. Sample Collection and Measurement of Physical Parameters

Each pond was sampled twice at one week interval between the end of January and early February 2010, except for CTA ponds which were sampled only once in May 2010. Water physical and chemical parameters, including water depth (m), temperature (°C), dissolved oxygen (mg O_2_ L^−1^), conductivity (μS), pH, nitrate, ammonium and ammonia concentrations were measured with hand-held probes (Yellow Springs Instruments Inc., Yellow Springs, OH, USA). Water transparency was measured with a Secchi disc, and water samples were collected to measure chlorophyll-*a* biomass (μg Chl-*a* L^−1^; particle size fraction < 125 μm) and dissolved phosphorus content (μg P L^−1^). Chl-*a* was determined using the fluorimetric method proposed by Yenstch and Menzel and adapted by Holm-Hansen and Rieman for the extraction in methanol [95,96]. Water filtered by 0.45 μm membranes (Millipore Co., Billerica, MA, USA) was analyzed for orthophosphate by the ascorbate-molybdate method [97].

Samples to assess the microbial community were collected from both the water column (2 mL) and sediments (1.5 g wet weight) and kept at 10 °C until inoculation of plates in the laboratory. Similar samples for bacterial enumeration in water and soil were collected and transported at −20 °C on a Nalgene^®^ Labtop cooler (Thermo Fisher Scientific, Inc., Waltham, MA, USA) and stored at −80 °C upon arrival at the laboratory. Simultaneously, 500 mL of water from the pond was collected in a sterilized bottle and 150–250 mL were filtered onto sterile 25 mm GF/F filters (0.7 μm nominal pore size; Whatman^®^, Sigma-Aldrich, St. Louis, MO, USA) using sterilized syringes and filter-holders. The filters were placed on eppendorfs, and transported in a Nalgene^®^ Labtop cooler at −20 °C to the laboratory where they were stored at −80 °C. Lipid extraction and analysis was performed within 2 days of collection. 

For microscopic quantification of bacterial cells, 2 mL duplicates of diluted samples (1:10; 1:100; 1:1000) were taken from the water collected in each pond and SYTO 9 nucleic acid stain (Molecular Probes, Life Technologies, Thermo Fisher Scientific Inc., Waltham, MA, USA) was added at a concentration of 3 µL/mL to stain all bacteria. Soil samples (0.5 g) were mixed with 5 mL of sterilized PBS, stirred for 2 h, sonicated for 1 min over ice and diluted in PBS (1:10; 1:100; 1:1000). Both types of samples (from water column and soil) were concentrated on 0.2 µm sterilized polycarbonate membrane filters (Millipore) using a stericup vacuum filtration system (Millipore) and observed by fluorescence microscopy using an Olympus CX40 microscope, with an Olympus U-RFL-T burner and an U-MWB mirror cube unit (excitation filter: BP450-480; barrier filter: BA515). At least 50 images, of areas chosen randomly at approximately even intervals between the periphery and the centre of the filter, were captured by an Evolution™ MP5.1 CCD colour camera using the software Image-Pro Plus (both from Media Cybernetics, Inc., Rockville, MD, USA). Green cells were counted using the same software. 

On the first sampling date, triplicate zooplankton samples were collected using a plankton net (75 μm mesh size) to make horizontal tows of 2–4 m length from the deep centre to the margin of the pond. Previous studies indicated that for ponds with less than 1.5 m depth, horizontal hauls at ≈0.5 m depth were ideal to sample all crustacea [21]. The horizontal hauls were made from the centre of the pond to the vertices of an imaginary triangle fitting the pond. Zooplankton were anaesthetised in carbonated water, fixed in sugar saturated formaldehyde (2%) and stored in glycerinated 70% ethanol for microscopic identification. Identification of copepods followed [98,99] and [100], and the identification of cladocerans followed [101] and [102]. The presence of at least one species characteristic of Mediterranean temporary pond ecosystems [21,102,103,104] conditioned the selection of ponds to inspect the contribution of matter of heterotrophic origin to the planktonic food web: *Daphnia (Ctenodaphnia) hispanica* (Glagolev and Alonso, 1990), *Moina brachiata* (Jurine, 1820), *Diaptomus kenitraensis* (Kiefer, 1926), *Dussartius baeticus* (Dussart, 1967) *Hemidiaptomus roubaui* (Richard, 1988) or large branchiopod species e.g., *Chirocephalus diaphanus* (Prévost 1803) and *Branchipus cortesi* (Alonso and Jaume 1991). All zooplankton species were observed to exhibit filter feeding behaviour although the calanoid *H. roubaui* exhibit the ability to attack and prey on smaller zooplankton. On the second sampling date, large zooplankton species (*D. hispanica*, *Simocephalus* sp., *H. roubaui*, *C. diaphanus* and *B. cortesi*) were pipetted out of the samples after collection and placed in groups of 20 individuals in 0.5 L transporting flasks containing pond water filtered through a 20 μm mesh sieve (Retsch, Verder Scientific, Haan, Germany). The remaining zooplankton samples were placed within 5 L plastic vessels containing pond water filtered through a 20 μm mesh sieve. Samples were kept in a cool box for transportation to the laboratory. In the laboratory, animals were sorted under a dissecting microscope (Leica AG, Wetzlar, Germany), washed three times in water filtered through GF/F glass filters (Whatman^®^, Sigma-Aldrich) and placed in filtered water for 4 h at 10 °C to empty their guts. Animals were then washed once in filtered pond water and gently filtered onto GF/A glass filters and immediately frozen at −80 °C. Filters containing seston and animals were stored at −80 °C until lipid extraction and fractioning.

### 3.2. Lipid Extraction and Fractioning

Total lipids were extracted from the filters by a modified Bligh and Dyer method [105], according to Findlay and co-workers [28], using a mixture of chloroform and methanol (1:2, v/v). Phase separation was favoured by further addition of chloroform and water (to a final solvent composition of 1:1:0.5, v/v). The chloroform layer containing the total lipids was collected, the chloroform was evaporated on a vacuum evaporation system (RapidVap from Labconco, Kansas City, MO, USA) and the lipids were dissolved in 0.5 mL of fresh chloroform. The lipids were fractioned into neutral-, glyco- and phospholipids on a heat-activated silicic acid column (Merck, Darmstadt, Germany) by sequential elution with chloroform, acetone and methanol. Fatty acid methyl esters (FAMEs) were obtained from apolar fractions by derivatisation with H_2_SO_4_-methanol and by mild alkaline transmethylation with methanolic NaOH from the polar lipid fraction. C19:0 (Sigma, St. Louis, MO, USA) was used as internal standard to allow fatty acid quantification. FAMEs were analysed on a 6890N gas chromatograph from Agilent Technologies (Palo Alto, CA, USA), with a flame ionization detector and a 7683B series injector, equipped with a 25 m long Agilent J&W Ultra 2 capillary column from Agilent. FAME were identified by the PLFAD1 method of Sherlock^®^ software version 6.2 from MIDI, Inc (Newark, DE, USA) and also by using a qualitative standard of bacterial fatty acid methyl esters and one of polyunsaturated fatty acids, both from Supelco, and a methyl *cis*-11-octadecenoate standard solution from Sigma-Aldrich. Peak identification was confirmed by injecting both standard and selected samples on a gas chromatograph mass spectrometer (5975B inlet MSD from Agilent) equipped with a DB-1 column from J&W Scientific.

### 3.3. Data Analysis

The heterotrophic/autotrophic contribution to the lower level of the food web of the Mediterranean temporary ponds was determined using phospholipid fatty acid data analyzed by the matrix-factorization program CHEMTAX [37]. This program uses factor analysis and a steepest descent algorithm to determine, the best fit of the experimental data on to an initial fatty acid ratio (input) matrix. The software was designed to determine the proportion of pigments attributed to a given phytoplankton group in the community based on a given input matrix of pigment ratios. Using an interactive process for a certain input matrix, the software optimizes the pigment ratio for each group and uses the final calculated ratio to determine the proportion of the pigment in each group present in the community. CHEMTAX has also been successfully used to study microbial communities using phospholipids-derived fatty acids [24,27,106]. 

In the present study, CHEMTAX version 1.95 was kindly provided by the CHEMTAX authors. As suggested, the initial fatty acid input ratios were determined using the ratios measured from regionally specific taxon grown under experimental conditions. The fatty acid profile of each bacterial phylum was obtained from the average of the bacterial genera isolated from samples collected on the studied temporary ponds. Generic algae and fungi fatty acid profiles of the observed phyla resulted from experimental and published data [30,44,107,108]. 

Since CHEMTAX is sensitive to the values of the initial ratio matrix, ratio optimization was performed using the methods suggested by Latasa [75] and Wright *et al.* [73].

The bacterial species richness measured as the cumulative number of bacterial species identified in each pond on both sampling dates was correlated (Spearman’s rank correlation) to the total FA content of seston, colony forming units, chlorophyll-*a* concentration and total dissolved phosphorus (dP).

Principal component analysis (PCA) was applied to data to inspect if the ordination of samples (zooplankton in the several ponds) could be related to the pattern of FA composition of zooplankton reserve lipids to that of the seston. PCA is a method that indicates (indirect) gradients by producing a smaller set of variables (principal components) that explain the variability of a larger set of variables ([109]; IBM SPSS Statistics 20.0, Armonk NY, USA). In order to reduce the large number of FA (variables) identified in relation to sample size, FA with <14 carbons were removed from the analysis because they may arise from *de novo* biosynthesis in the consumer and have no relation to diet intake (see [45]). FA variables that did not meet the Kaiser-Meyer-Olkin measure of sampling adequacy (>0.50) and communality (>0.50) were also removed from the analysis. As PCA analysis is based on the assumption of variance homogeneity, data were scaled (*i.e*., divided by standard deviation) prior to PCA analyses.

#### Microbial Groups

Samples from both the water column (1 mL in triplicate) and sediments (*ca.* 1 g in triplicate) of each temporary pond were collected and stored at 10 °C until inoculation of Tryptic Soy Agar (TSA) plates. The inoculation of the plates was done with 100 μL of liquid sample or 100 μL of mineral medium used to extract the cells from the sediments to allow both the determination of the CFU and the isolation of the microorganisms. To favour the growth of slow-growing organisms in the samples, low nutrient concentration was tested simultaneously as suggested [47] by inoculating 10- and 100-diluted TSA plates. All plates were incubated at 30 °C for 24 h and photographed with a Nikon Coolpix P5100 to help the visual identification of the colonies as bacteria, algae, or fungi. Each bacterial isolated colony was used to inoculate a new TSA plate for identification using the Sherlock^®^ Microbial ID System from MIDI, Inc. Briefly, the plates containing each isolate were grown at 30 °C for 24 h ± 1 h, when the exponentially growing cells were harvested, and their fatty acids were extracted and methylated to FAMES using the Instant FAME procedure according to the instructions provided by MIDI. FAMEs were analyzed on the gas chromatograph described previously. The original plates were further examined after 48 and 72h to assess the existence of fastidious species and again photographed. Samples which could not be identified by Sherlock^®^ were further studied to assess the existence of known fatty acid biomarkers and observed under the microscope (CX40 from Olympus, Japan) for educated guessing. 

Cells from the identified species were grown overnight at 20 °C on sterilized water brought from the ponds in 96-well plates and the fatty acids were extracted and fractioned as described in Section 3.2 to determine the generic PLFA profile of the respective phylum. The fungi and algae on the samples were identified by direct observation and microscopy images and the PLFA used for the corresponding phylum was based on the experimental data obtained in this study and that found on published literature, respectively, as mentioned.

## 4. Conclusions

Temporary Mediterranean ponds present important bacterial and fungal communities which associated to detritus of autochthonous or allochthonous origin may render them largely heterotrophic. This study demonstrates that PLFA may be used to inspect the composition of the plankton microbial community and that the heterotrophic energy is channeled into the zooplankton. Although zooplankton may require fatty acids from autotrophic sources for their growth and development, they have the ability to deposit FA of bacterial origin into both their reserve and structural lipids. The role of bacterial FA in aquatic food webs is far from clear, yet its incorporation into the tissues of zooplankton suggests that they may be an important source of energy in environments where autotrophs of high nutritional quality are available in limited quantity.

## Supplementary Materials

Supplementary materials can be accessed at: http://www.mdpi.com/1420-3049/19/5/5570/s1.
