# Supplementary material for: Fatty Acids as a Tool to Understand Microbial Diversity and Their Role in Food Webs of Mediterranean Temporary Ponds
Source: Molecules. 2014 Apr 30;19(5):5570–98. doi: 10.3390/molecules19055570 (PMC6271346; doi:10.3390/molecules19055570)
Supplement: Supplementary file 1 [file molecules-19-05570-s001.pdf]

Supplementary Materials

Table S1. Fatty acid (FA) composition of seston (fraction < 125 μm) and zooplankton crustacea in Torrão pond. Reserve lipids are the sum of apolar and acetone mobile lipid fractions plus free fatty acids.

Torrao	Filter	**Cladocera**						**Large Branchiopods**	
		***Simocephalus* sp.**		***Ceriodaphnia quadrangula***		***Daphnia hispanica***		***Chirocephalus diaphnanus***	
		**Reserve lipids**	**Phospho-lipids**	**Reserve lipids**	**Phospho-lipids**	**Reserve lipids**	**Phospho-lipids**	**Reserve lipids**	**Phospho-lipids**
10:0	0.00	0.32	0.26	2.54	0.00	0.92	0.00	0.40	0.04
12:0	2.37	3.61	1.15	3.98	0.00	3.84	2.01	4.01	0.26
14:0	7.75	2.40	1.10	2.34	0.71	2.50	0.73	2.19	0.61
16:0	19.36	5.86	12.12	9.00	7.32	7.91	8.52	7.21	10.98
18:0	16.47	1.38	4.59	4.56	1.66	2.93	3.20	3.45	5.49
∑ Saturated (without 15:0)	50.28	13.87	20.24	22.41	10.26	19.61	15.38	19.65	18.50
15:1ω8	0.00	0.00	0.00	1.59	0.20	1.08	0.00	0.45	0.00
16:1ω4 (Diatoms)	0.00	0.00	0.00	0.00	0.00	0.13	0.00	0.13	0.00
16:1ω5 (Fungi)	0.78	0.39	0.38	0.21	0.08	0.89	0.23	0.73	0.55
16:1ω7 *	2.79	19.99	8.97	10.70	34.73	13.01	11.63	17.58	7.16
16:1ω9 *	0.50	0.00	0.00	0.00	0.00	2.37	4.01	0.41	0.00
18:1ω7 *	3.15	0.00	5.42	0.74	1.52	0.31	7.07	0.10	3.23
18:1ω9 *	13.03	0.14	10.67	2.33	5.88	2.07	9.46	4.92	25.12
∑ Mono-unsaturated	20.26	31.25	36.27	28.34	44.66	34.69	35.43	33.65	38.83
∑ Bacterial FA	22.75	8.95	8.95	13.99	13.99	17.20	17.20	14.42	14.42
16:2ω4	0.43	0.00	0.00	0.00	0.00	0.00	0.00	0.00	0.00
16:3ω4	2.07	0.00	0.00	0.00	0.00	0.00	0.00	0.00	0.00
16:3ω6	0.00	0.00	0.00	0.00	0.00	0.00	0.00	0.00	0.00
18:2ω6	4.02	1.78	5.58	1.20	0.69	2.15	2.67	1.97	4.92
18:3ω3	1.20	28.99	11.35	16.00	8.20	14.76	20.36	15.54	3.11
20:4ω6	1.08	0.88	6.53	0.13	0.97	0.75	3.21	1.02	5.86
20:5ω3	2.34	2.22	11.07	0.52	4.60	2.57	8.47	3.93	20.55
22:5ω3	0.11	0.12	0.00	0.72	0.17	0.57	0.20	1.15	0.29
22:6ω3	1.50	0.00	1.20	0.10	0.27	0.13	0.11	0.52	2.15
∑ PUFA	12.76	33.99	35.74	18.68	14.90	20.93	35.02	24.12	36.89
∑ C>24	0.00	0.00	0.00	0.66	0.00	0.81	4.50	1.43	0.57
∑ ω3	5.31	31.33	31.33	17.35	17.35	18.03	18.03	21.13	21.13
ω3/ω6	1.04	11.78	11.78	13.01	13.01	6.22	6.22	7.07	7.07

* includes FA measured as DMA, likely to resulting from vinyl bonds with glycerol. Bacterial FA are the sum of iso-, antiso, 10-Methyl and cyclo FA, 15:0, and 16:1ω8.

Table S2. Fatty acid (FA) composition of seston (fraction < 125 µm) and zooplankton crustacea in Bunho pond. Reserve lipids are the sum of apolar and acetone mobile lipid fractions plus free fatty acids.

**Bunho**	**Filter**	**Cladocera**							
		***Simocephalus* sp.**		***Moina brachiata***		***Ceriodaphnia reticulata***		***Daphnia hispanica***	
		**Reserve lipids**	**Phospho-lipids**	**Reserve lipids**	**Phospho-lipids**	**Reserve lipids**	**Phospho-lipids**	**Reserve lipids**	**Phospho-lipids**
10:0	0.00	0.00	0.00	0.00	0.00	0.00	0.00	0.27	0.23
12:0	2.05	6.53	0.00	7.23	0.00	5.58	0.00	5.88	1.08
14:0	5.51	2.20	0.94	3.35	1.02	3.30	0.93	2.73	1.55
16:0	18.17	6.87	15.55	14.69	12.79	14.43	15.90	12.32	15.16
18:0	5.10	5.78	5.31	8.25	7.61	5.75	6.65	6.07	7.43
∑ Saturated (without 15:0)	35.64	22.88	22.91	34.96	22.99	29.06	24.81	28.53	28.33
15:1ω8	0.00	1.89	0.00	1.03	0.00	1.75	0.00	1.50	0.00
16:1ω4 (Diatoms)	0.00	0.48	0.00	0.00	0.00	0.00	0.00	0.17	0.00
16:1ω5 (Fungi)	0.68	0.39	0.00	0.00	0.38	1.20	0.28	0.61	0.20
16:1ω7 *	2.39	11.63	4.44	5.83	8.23	16.85	7.06	10.11	6.04
16:1ω9 *	15.92	0.26	0.00	3.20	0.00	0.00	1.61	0.00	3.30
18:1ω7 *	12.55	0.25	0.00	0.00	8.36	0.00	4.89	0.08	1.83
18:1ω9 *	6.59	3.35	14.81	8.51	11.99	4.69	22.43	3.88	15.51
∑ Mono-unsaturated	40.84	32.87	30.80	31.03	31.03	29.96	37.35	28.55	31.38
∑ Bacterial FA	36.78	22.05	22.05	12.25	12.25	18.99	18.99	15.50	15.50
16:2ω4	0.00	0.00	0.00	0.00	0.00	0.00	0.00	0.00	0.00
16:3ω4	0.00	0.00	0.00	0.00	0.00	0.00	0.00	0.00	0.00
16:3ω6	0.00	0.00	0.00	0.66	0.00	0.54	0.00	0.20	0.00
18:2ω6	5.20	1.83	5.22	2.31	5.43	4.57	7.63	3.47	4.39
18:3ω3	3.11	7.14	1.11	10.75	1.21	6.68	1.36	7.89	1.38
20:4ω6	1.95	0.52	6.31	1.59	9.16	1.20	7.41	1.05	5.52
20:5ω3	3.49	0.97	12.67	2.95	17.25	2.70	14.51	3.81	15.05
22:5ω3	0.00	3.16	0.00	4.91	0.64	3.82	0.28	2.92	0.60
22:6ω3	1.29	0.27	2.72	0.00	1.83	1.02	2.19	1.27	6.38
∑ PUFA	15.03	13.89	28.04	23.16	35.51	20.51	33.38	20.60	33.33
∑ C>24	0.00	2.02	17.28	0.92	0.00	0.00	0.00	3.09	1.69
∑ω3	7.89	11.54	11.54	18.61	18.61	14.22	14.22	15.88	15.88
ω3/ω6	1.10	4.91	4.91	4.09	4.09	2.26	2.26	3.37	3.37

* includes FA measured as DMA, likely to resulting from vinyl bonds with glycerol. Bacterial FA are the sum of iso-, antiso, 10-Methyl and cyclo FA, 15:0, and 16:1ω8.

Table S3. Fatty acid (FA) composition of seston (fraction < 125 µm) and zooplankton crustacea in Sector A pond. Reserve lipids are the sum of apolar and acetone mobile lipid fractions plus free fatty acids.

		**Cladocera**		**Large Branchiopods**			
		Ceriodaphnia reticulata		***Chirocephalus diaphnanus***		***Branchipus cortesi***	
**Sector A**	**Filter**	**Reserve lipids**	**Phospho-lipids**	**Reserve lipids**	**Phospho-lipids**	**Reserve lipids**	**Phospho-lipids**
10:0	0.00	0.67	0.00	3.09	0.00	1.84	0.00
12:0	2.64	8.68	1.04	3.96	1.07	3.21	0.41
14:0	4.75	2.27	2.26	1.55	0.59	1.19	0.28
16:0	26.47	11.25	15.20	5.59	10.69	5.72	4.42
18:0	15.53	6.50	6.44	2.41	3.90	1.88	2.26
∑ Saturated (without 15:0)	50.39	35.53	26.26	17.45	17.27	14.18	7.78
15:1ω8	0.00	1.54	0.00	1.29	0.00	0.72	0.00
16:1ω4 (Diatoms)	0.00	0.00	0.00	0.00	0.00	0.00	0.00
16:1ω5 (Fungi)	2.71	0.00	0.00	1.20	0.41	0.89	0.27
16:1ω7 *	6.14	7.66	5.52	13.91	12.54	14.26	17.64
16:1ω9 *	0.00	2.69	0.00	0.19	0.00	0.00	0.00
18:1ω7 *	5.04	0.00	6.30	0.06	6.37	0.00	4.34
18:1ω9 *	8.45	5.62	7.59	4.27	16.43	6.51	9.07
∑ Mono-unsaturated	22.35	21.21	25.37	35.93	38.82	34.19	42.30
∑ Bacterial FA	18.67	12.24	12.24	15.85	15.85	14.60	14.60
16:2ω4	0.00	0.00	0.00	0.00	0.00	0.00	0.00
16:3ω4	3.00	0.00	0.00	0.00	0.00	0.00	0.00
16:3ω6	0.00	0.00	0.00	0.20	0.00	0.27	0.00
18:2ω6	3.50	2.33	4.73	2.11	3.72	2.08	2.12
18:3ω3	0.00	10.23	1.96	15.77	12.97	16.48	4.54
20:4ω6	0.00	0.00	8.29	1.12	5.78	1.26	5.69
20:5ω3	1.87	1.41	16.48	2.80	12.08	2.88	8.68
22:5ω3	0.00	4.27	0.00	0.34	0.00	0.23	0.00
22:6ω3	0.00	0.40	10.67	0.64	0.79	0.46	0.38
∑ PUFA	8.36	18.63	42.14	22.98	35.34	23.66	21.42
∑ C>24	0.00	2.03	0.00	0.50	0.00	6.79	0.00
∑ω3	1.87	16.30	16.30	19.54	19.54	20.05	20.05
ω3/ω6	0.36	6.98	6.98	5.69	5.69	5.56	5.56

* includes FA measured as DMA, likely to resulting from vinyl bonds with glycerol. Bacterial FA are the sum of iso-, antiso, 10-Methyl and cyclo FA, 15:0, and 16:1ω8.

Table S4. Fatty acid (FA) composition of seston (fraction < 125 µm) and zooplankton crustacea in Sector B pond. Reserve lipids are the sum of apolar and acetone mobile lipid fractions plus free fatty acids.

		**Cladocera**				**Large Branchiopods**	
		***Simocephalus* sp.**		***Daphnia hispanica***		***Chirocephalus diaphnanus***	
**Sector B**	**Filter**	**Reserve lipids**	**Phospho-lipids**	**Reserve lipids**	**Phospho-lipids**	**Reserve lipids**	**Phospho-lipids**
10:0	0.00	0.16	0.13	0.59	0.11	0.07	0.04
12:0	13.44	5.07	0.57	4.86	1.54	3.47	0.25
14:0	8.98	2.30	1.02	2.61	1.14	1.53	0.91
16:0	12.50	6.36	13.83	10.11	11.84	6.36	12.28
18:0	13.76	3.58	4.95	4.50	5.31	3.23	5.00
∑ Saturated (without 15:0)	48.68	18.38	21.58	24.07	21.86	15.75	19.71
15:1ω8	0.00	0.94	0.00	1.29	0.00	0.77	0.00
16:1ω4 (Diatoms)	0.00	0.24	0.00	0.15	0.00	0.12	0.00
16:1ω5 (Fungi)	3.40	0.39	0.19	0.75	0.22	0.74	0.48
16:1ω7 *	3.20	15.81	6.71	11.56	8.83	15.87	4.54
16:1ω9 *	9.97	0.13	0.00	1.19	3.65	0.32	0.00
18:1ω7 *	5.48	0.13	2.71	0.19	4.45	0.00	0.00
18:1ω9 *	3.69	1.75	12.74	2.97	12.48	4.96	29.24
∑ Mono-unsaturated	37.22	32.06	33.53	31.62	33.40	35.33	35.99
∑ Bacterial FA	21.14	15.50	15.50	16.63	16.63	14.14	14.14
16:2ω4	0.00	0.00	0.00	0.00	0.00	0.00	0.00
16:3ω4	4.83	0.00	0.00	0.00	0.00	0.00	0.00
16:3ω6	0.00	0.00	0.00	0.10	0.00	0.25	0.00
18:2ω6	0.00	1.80	5.40	2.81	3.53	2.16	5.28
18:3ω3	12.35	18.07	6.23	11.32	10.87	15.94	4.38
20:4ω6	0.00	0.70	6.42	0.90	4.37	1.19	6.91
20:5ω3	0.00	1.60	11.87	3.19	11.76	3.37	19.22
22:5ω3	1.51	1.64	0.00	1.74	0.40	0.54	0.14
22:6ω3	0.00	0.13	1.96	0.70	3.25	0.60	2.89
∑ PUFA	18.68	23.94	31.89	20.77	34.18	24.04	38.83
∑ C>24	0.00	1.01	8.64	1.95	3.09	1.49	0.12
∑ω3	13.85	21.43	21.43	16.96	16.96	20.44	20.44
ω3/ω6	1.73	8.35	8.35	4.79	4.79	5.68	5.68

* includes FA measured as DMA, likely to resulting from vinyl bonds with glycerol. Bacterial FA are the sum of iso-, antiso, 10-Methyl and cyclo FA, 15:0, and 16:1ω8.

Table S5. Fatty acid (FA) composition of seston (fraction < 125 µm) and zooplankton crustacea in Poste pond. Reserve lipids are the sum of apolar and acetone mobile lipid fractions plus free fatty acids.

		**Large Branchiopods**	
		Tanymastix stagnales	
**Poste**	**Filter**	**Reserve lipids**	**Phospho-lipids**
10:0	0.00	4.29	0.00
12:0	2.06	5.09	1.71
14:0	4.82	1.04	0.72
16:0	14.44	2.98	5.67
18:0	18.00	2.54	2.34
∑ Saturated (without 15:0)	40.32	17.58	10.89
15:1ω8	0.00	1.31	0.00
16:1ω4 (Diatoms)	0.00	0.94	0.00
16:1ω5 (Fungi)	3.60	0.00	0.45
16:1ω7 *	0.00	9.21	21.44
16:1ω9 *	0.00	1.60	0.00
18:1ω7 *	4.27	0.16	4.63
18:1ω9 *	8.76	1.91	3.81
∑ Mono-unsaturated	31.03	28.99	33.91
∑ Bacterial FA	28.69	18.09	18.09
16:2ω4	0.65	0.00	0.00
16:3ω4	0.00	0.00	0.00
16:3ω6	0.00	0.42	0.00
18:2ω6	7.74	0.86	1.97
18:3ω3	0.00	20.21	32.20
20:4ω6	0.00	0.00	2.67
20:5ω3	1.56	0.22	6.52
22:5ω3	0.00	0.39	0.00
22:6ω3	0.00	0.00	0.40
∑ PUFA	9.95	22.10	43.76
∑ C>24	0.00	3.91	0.00
∑ω3	1.56	20.82	20.82
ω3/ω6	0.20	16.34	16.34

* includes FA measured as DMA, likely to resulting from vinyl bonds with glycerol. Bacterial FA are the sum of iso-, antiso, 10-Methyl and cyclo FA, 15:0, and 16:1ω8.
